# Low RBC counts predict high on-treatment platelet reactivity in patients undergoing percutaneous coronary intervention and treated with clopidogrel

**DOI:** 10.7555/JBR.37.20230035

**Published:** 2023-12-15

**Authors:** Qian Gu, Qin Wang, Rui Hua, Wenhao Zhang, Jianzhen Teng, Jiazheng Ma, Zhou Dong, Xiaoxuan Gong, Chunjian Li

**Affiliations:** Department of Cardiology, the First Affiliated Hospital of Nanjing Medical University, Nanjing, Jiangsu 210029, China

Dear Editor,

Cardiovascular disease is the leading cause of deaths worldwide, with coronary artery disease (CAD) accounting for approximately 50% of its mortality. Dual antiplatelet therapy, including aspirin and a P2Y_12_ inhibitor, is the most important treatment for CAD patients undergoing percutaneous coronary intervention (PCI) to prevent recurrent ischemic events and cardiac death. Clopidogrel is one of the commonly used P2Y_12_ inhibitors. However, up to 30% of patients treated with a standard dose of clopidogrel present with high on-treatment platelet reactivity (HOPR), which is associated with the increased ischemic risks^[1]^. The causes of HOPR are multifactorial and complex. Polymorphisms of cytochrome P450 enzyme genes (such as *CYP2C19*) have been widely reported to influence platelet response to clopidogrel^[2]^, which, however, may account for only 12% of HOPR^[2]^. The etiology for the rest of the patients exhibiting HOPR remains uncertain, which is a residual ischemic risk for CAD patients who are taking clopidogrel. The present study aims to investigate risk factors associated with HOPR in CAD patients undergoing PCI and receiving the dual antiplatelet therapy with aspirin and clopidogrel. The present study is a cross-sectional cohort study performed in the First Affiliated Hospital of Nanjing Medical University using our pre-registered database (Unique Identifier: NCT01968499), complied with the Helsinki declaration and local regulations, and was approved by the Institutional Review Board of the First Affiliated Hospital of Nanjing Medical University (No. 2011-SRFA-099). A written informed consent was obtained from each patient.

CAD patients who had undergone PCI and taken clopidogrel (75 mg/day) combined with aspirin (100 mg/day) for more than five days were consecutively enrolled between April 2011 and October 2016 in the coronary care unit of the First Affiliated Hospital of Nanjing Medical University. We excluded patients who were: (1) intolerant to aspirin or clopidogrel; (2) with hematological diseases; (3) with baseline hemoglobin < 90 g/L, or platelet count < 80 × 10^9^/L or > 450 × 10^9^/L; (4) taking other antiplatelet agents or anticoagulants or any drugs that could potentially interfere with the antiplatelet efficacy of the study drugs; and (5) with end-stage diseases (*e.g.*, cancer) or other conditions that were inappropriate to be recruited at the discretion of the investigators. The patients' demographics, present disease history, past disease history, personal history, physical examination, laboratory examination, and medications were recorded. In addition, venous blood was collected into two 2.7 mL vacutainer tubes containing 3.2% sodium citrate two hours after the patients took clopidogrel and aspirin. Platelet reactivities were measured by the light transmission aggregometry within two hours of the sampling. Platelet-rich plasma (PRP) was separated by centrifuging the blood sample at 200 *g* at 22 ℃ for 5 min, and platelet poor plasma (PPP) was obtained by spinning the remaining blood at 2465 *g* for another 10 min. Platelet counts were adjusted by adding PPP to PRP to achieve a count of 250 × 10^9^/L. A total of 500 μL adjusted PRP was tested by a Chronolog aggregometer (Model 700, Chrono-log Corporation, Havertown, PA, USA) with 500 μL PPP as control. Platelet aggregation was induced by 2.5 μL adenosine diphosphate (ADP) with a final concentration of 5 μmol/L or 10 μL arachidonic acid (AA) with a final concentration of 1 mmol/L, and recorded as PL_ADP_ or PL_AA_, respectively. HOPR was defined as PL_ADP_ > 40%^[3]^.

SPSS version 25.0 (SPSS, Inc., Chicago, IL, USA) was used for statistical analysis. Continuous variables were presented as mean ± standard deviation, and categorical variables were expressed as frequencies or percentages. The independent Student's *t-*test or Chi-square test was used as appropriate to assess differences between the HOPR and non-HOPR groups, and the variables that were significantly different between the two groups were included in the logistic regression analysis to identify the factors associated with the HOPR. Covariates with *P*-values less than 0.05 in univariable regression analysis were selected for the inclusion in the multiple logistic regression model. A two-tailed *P-*value < 0.05 was considered statistically significant for all the tests.

As a result, 1649 eligible patients were included in the analyses. By in platelet reactivity assessment, HOPR was observed in 389 (23.6%) patients. The baseline characteristics of patients are listed in ***Table 1***.

**Table 1:** Baseline characteristics of patients with or without HOPR

Variables	HOPR			Non-HOPR		*P*-value
	*N*	[*n *(%)] or (mean±SD)		*N*	[*n *(%)] or (mean±SD)	
Female	389	117 (30.1)		1260	294 (23.3)	0.007
Age (years)	389	64.4±10.3		1260	63.6±10.5	0.201
BMI (kg/m^2^)	373	25.0±3.0		1182	24.6±3.1	0.023
Smoking	387	160 (41.3)		1251	600 (48.0)	0.023
Drinking	386	80 (20.7)		1248	314 (25.2)	0.075
Hypertension	388	260 (67.0)		1258	831 (66.1)	0.728
Diabetes mellitus	387	100 (25.8)		1253	331 (26.4)	0.822
Hyperlipidemia	330	33 (10.0)		852	81 (9.5)	0.797
PCI history	385	25 (6.5)		1249	127 (10.2)	0.030
RBC (10^12^/L)	384	4.4±0.5		1237	4.5±0.6	0.010
Hemoglobin (g/L)	384	134.1±15.3		1238	135.8±17.7	0.100
WBC (10^9^/L)	384	7.4±3.0		1238	6.9±2.4	0.004
Neutrophil ratio (%)	384	64.0±11.8		1236	63.4±10.1	0.398
Platelet (10^9^/L)	384	189.2±56.4		1238	195.2±63.4	0.094
ALT (U/L)	380	37.5±34.8		1242	36.3±50.3	0.660
LDH (U/L)	374	310.0±338.9		1216	265.1±255.9	0.018
γ-GGT (U/L)	344	44.2±65.0		1192	47.2±93.4	0.585
TBIL (µmol/L)	346	13.3±6.1		1200	13.6±24.9	0.849
DBIL (µmol/L)	345	4.2±2.1		1194	6.5±62.7	0.504
IBIL (µmol/L)	345	9.1±4.4		1189	8.6±4.5	0.087
BUN (mmol/L)	380	6.0±3.7		1238	8.0±41.1	0.347
Creatinine (µmol/L)	380	77.9±22.0		1240	80.7±40.8	0.209
Uric acid (µmol/L)	374	332.1±99.0		1221	346.1±93.8	0.013
FBG (mmol/L)	370	6.3±2.2		1194	6.0±2.0	0.027
HbA1c (%)	135	6.8±1.7		411	6.8±1.5	0.567
TC (mmol/l)	373	4.3±1.2		1221	4.3±2.1	0.911
TG (mmol/L)	374	1.6±0.9		1222	1.9±5.9	0.325
LDL-C (mmol/L)	374	2.6±0.9		1220	3.4±12.1	0.231
HDL-C (mmol/L)	374	1.1±0.3		1220	1.3±5.5	0.569
Lp(a) (mg/L)	367	280.4±249.7		1210	282.7±267.2	0.883
CK-MB (ng/mL)	312	39.1±79.1		968	36.4±138.7	0.750
PCT (ng/mL)	135	0.4±1.3		370	1.0±2.8	< 0.001
CRP (mg/L)	82	8.8±19.9		241	7.3±18.9	0.551
PT (s)	332	11.9±1.2		1163	11.9±1.4	0.578
APTT (s)	329	48.2±394.5		1160	26.7±9.9	0.323
INR	332	1.4±3.1		1160	1.1±1.7	0.129
PL_ADP_ (%)	389	50.4±7.7		1260	24.5±9.4	< 0.001
PL_AA_ (%)	389	5.2±9.8		1260	4.0±4.6	0.017
PPIs	389	72 (18.5)		1260	233 (18.5)	0.994
Statins	389	367 (94.3)		1260	1128 (89.5)	0.004
CCBs	389	136 (35.0)		1260	413 (32.8)	0.424
Diagnoses	377			1234		0.033
SA	69	69 (18.3)		305	305 (24.7)	
UA	187	187 (49.6)		605	605 (49.0)	
NSTEMI	34	34 (9.0)		88	88 (7.2)	
STEMI	87	87 (23.1)		236	236 (19.1)	
Abbreviations: HOPR, high on-treatment platelet reactivity; BMI, body mass index; PCI, percutaneous coronary intervention; RBC, red blood cell; WBC, white blood cell; ALT, alanine transaminase; LDH, lactate dehydrogenase; γ-GGT, gamma-glutamyl transpeptidase; TBIL, total bilirubin; DBIL, direct bilirubin; IBIL, indirect bilirubin; BUN, blood urea nitrogen; FBG, fasting blood glucose; HbA1c, hemoglobin A1c; TC, total cholesterol; TG, total triglyceride; LDL, low density lipoprotein; HDL, high density lipoprotein; Lp(a), lipoprotein (a); CK-MB, creatine kinase-MB; PCT, procalcitonin; CRP, C-reactive protein; PT, prothrombin time; APTT, activated partial thromboplastin time; INR, international normalized ratio; PL_ADP_, platelet aggregation induced by adenosine diphosphate; PL_AA_, platelet aggregation induced by arachidonic acid; PPIs, proton pump inhibitors; CCBs, calcium channel blockers; SA, stable angina; UA, unstable angina; NSTEMI, non-ST-elevation myocardial infarction; STEMI, ST-elevation myocardial infarction; SD, standard deviation.						

Female, body mass index (BMI), smoking, PCI history, red blood cell (RBC) counts, white blood cell counts, lactate dehydrogenase, uric acid, fasting blood glucose, procalcitonin, PL_AA_, statin consumption, and CAD diagnosis were significantly associated with HOPR in the univariable logistic regression analyses (all *P* < 0.05) (***Table 2***). However, multivariable logistic regression analysis showed that only RBC count, BMI, and statin consumption were independently associated with HOPR (OR = 0.480, 95% CI: 0.302–0.763, *P* = 0.002; OR = 1.140, 95% CI: 1.054–1.232, *P* = 0.001; OR = 4.504, 95% CI: 1.004–20.208, *P* = 0.049, respectively) (***Table 2***).

**Table 2:** Logistic regression analysis for HOPR

Variables	*n*	Univariate logistic regression			Multivariate logistic regression	
		OR (95% CI)	*P*-value		OR (95% CI)	*P-*value
Female *vs.* male	389 (117/272)	1.413 (1.097–1.820)	0.007			
BMI^a^	373	1.044 (1.006–1.084)	0.023		1.140 (1.054–1.232)	0.001
Smoking *vs.* no smoking	387 (160/227)	0.765 (0.607–0.963)	0.023			
PCI history *vs. *no PCI history	385 (25/360)	0.614 (0.393–0.957)	0.031			
RBC^a^	384	0.757 (0.613–0.934)	0.010		0.480 (0.302–0.763)	0.002
WBC^a^	384	1.070 (1.026–1.116)	0.002			
LDH^a^	374	1.001 (1.000–1.001)	0.007			
Uric acid^a^	374	0.998 (0.997–1.000)	0.013			
FBG^a^	370	1.062 (1.009–1.119)	0.023			
PCT^a^	135	0.848 (0.736–0.977)	0.022			
Statins *vs. *no statins	389 (367/22)	1.952 (1.224–3.112)	0.005		4.504 (1.004–20.208)	0.049
SA	69	1	–			
UA *vs.* SA	187/69	1.366 (1.004–1.860)	0.047			
NSTEMI *vs.* SA	34/69	1.708 (1.063–2.744)	0.027			
STEMI *vs.* SA	87/69	1.630 (1.138–2.333)	0.008			
^a^The ORs for the continuous variables indicate that 1-unit increase of the variables is associated with a [(OR-1)×100] % increased risk of HOPR.Abbreviations: HOPR, high on-treatment platelet reactivity; OR, odds ratio; CI, confidence interval; BMI, body mass index; PCI, percutaneous coronary intervention; RBC, red blood cell; WBC, white blood cell; LDH, lactate dehydrogenase; FBG, fasting blood glucose; PCT, procalcitonin; SA, stable angina; UA, unstable angina; NSTEMI, non-ST-elevation myocardial infarction; STEMI, ST-elevation myocardial infarction.						

This is the first study to show that in CAD patients undergoing PCI and treated with clopidogrel, the RBC counts were independently and negatively associated with HOPR. Karolczak *et al*^[4]^ also reported a negative association between RBC counts and PL_ADP_; however, their results were based on 251 volunteers without acute coronary syndrome, and the platelet activity was measured by multiplate impedance aggregometry. In contrast, the present study recruited a large number of CAD patients, adopted the gold standard light transmission aggregometry method, and was the first to reveal a negative association between RBC counts with HOPR.

The effects of RBC on platelet aggregation may be mediated by ADP and nitric oxide (NO). ADP is stored in RBC and promotes platelet aggregation by binding to the P2Y_12_ receptor on the platelet surface, further activating glycoprotein (GP) Ⅱb/Ⅲa^[4]^. By contrast, NO, produced in the membrane and cytoplasm of RBC by the endothelial-type nitric oxide synthase (eNOS)^[4]^, has been reported to inhibit the activation of GP Ⅱb/Ⅲa and platelet aggregation *via* the increase of cyclic guanosine monophosphate (cGMP) and cyclic adenosine monophosphate (cAMP)^[5]^. One study demonstrated that during platelet aggregation, the inhibitory effect of NO predominated over the activating effect of ADP^[4]^. In addition, studies have confirmed that RBC plays an important role in maintaining cardiovascular homeostasis and vascular function^[6]^. RBC is responsible for the synthesis and release of NO *via* the release of adenosine triphosphate (ATP) to activate the endothelial purinergic receptors^[6]^. ATP is degraded to ADP and adenosine by nucleotidases. Both ATP and ADP are present in approximately equal amount in platelet granules, while RBC releases 10 times more ATP than ADP^[7]^. Moreover, RBC is involved not only in the ATP release, but also in regulating adenosine uptake. Therefore, we hypothesize that patients with higher RBC counts produce more NO and ATP, which causes a stronger inhibition of platelet aggregation and a less likelihood of HOPR. However, future studies are needed to clarify the mechanism of this association between RBC counts and HOPR.

Our results were also consistent with the reports indicating that BMI and statin consumption were independent risk factors for HOPR^[8–9]^. These may be explained by a decreased activity of CYP3A4 (a clopidogrel-related metabolic enzyme) and a relatively insufficient dose of clopidogrel in obese patients^[9]^. Most lipophilic statins, such as simvastatin and atorvastatin, are metabolized by the cytochrome P450 enzyme (mainly by CYP3A4) and competitively inhibit the metabolic activation of clopidogrel^[8]^. Thus, weight control is necessary for obese patients with CAD to reduce their risk of HOPR. Besides, lipid-lowering drugs that are less metabolized by CYP3A4 (*e.g.*, rosuvastatin) may be more suitable for patients with HOPR. Several clinical variables, such as WBC counts and procalcitonin, were reported to be associated with platelet reactivity^[10]^, but these associations were not confirmed in the present study. These may be explained by the biases from sample selection, sample size, differences in the detection methods, or different races of the study populations.

The present study has some potential limitations. Although there were independent correlations of RBC counts, BMI and statins consumption with HOPR, the differences between groups (patients with or without high platelet reactivity) were subtle. The clinical value of the observations needs to be further explored in future clinical trials.

In conclusion, the present study has revealed that low RBC counts, high BMI, and statin consumption may independently predict HOPR in CAD patients undergoing PCI and treated with clopidogrel.

The present study was supported by the National Natural Science Foundation of China (Grant No. 82170351), the Jiangsu Province's Key Provincial Talents Program (Grant No. ZDRCA2016013), and the Special Fund for Key R&D Plans (Social Development) of Jiangsu Province (Grant No. BE2019754).

Yours Sincerely,Qian Gu^△^, Qin Wang^△^, Rui Hua^△^, Wenhao Zhang, Jianzhen Teng, Jiazheng Ma, Zhou Dong, Xiaoxuan Gong^✉^, Chunjian Li^✉^ Department of Cardiology,the First Affiliated Hospital of Nanjing Medical University,Nanjing, Jiangsu 210029,China.^△^These authors contributed equally to this work.^✉^Chunjian Li and Xiaoxuan Gong. E-mails: lijay@njmu.edu.cn (Li) and xiaoxuangong@sina.com (Gong).
